# Specific serum antibody binding to phosphorylated and non-phosphorylated tau in non-cognitively impaired, mildly cognitively impaired, and Alzheimer’s disease subjects: an exploratory study

**DOI:** 10.1186/s40035-017-0100-x

**Published:** 2017-11-24

**Authors:** Andrea C. Klaver, Mary P. Coffey, David A. Bennett, David A. Loeffler

**Affiliations:** 1 Department of Neurology, Beaumont Research Institute, Beaumont Health, Royal Oak, MI USA; 2 Department of Biostatistics, Beaumont Research Institute, Beaumont Health, Royal Oak, MI USA; 3 Rush Alzheimer’s Disease Center, Chicago, IL 60612 USA

**Keywords:** Alzheimer’s disease, Antibodies, Elisa ELISA, Mild cognitive impairment, Phosphorylated tau

## Abstract

**Background:**

Tau vaccination and administration of anti-tau antibodies can prevent pathology and cognitive impairment in transgenic mouse models of tauopathy, suggesting that therapies which increase anti-tau antibodies might slow the development and/or progression of Alzheimer’s disease (AD). The extent to which individuals with no cognitive impairment (NCI) possess serum anti-tau antibodies, and whether their concentrations of these antibodies differ from anti-tau antibody levels in persons with mild cognitive impairment (MCI) or AD, are unclear. Previous studies measuring these antibodies did not account for antibody polyvalent binding, which can be extensive, nor that antibody binding to phosphorylated tau peptides could be due to binding to non-phosphorylated epitopes on those peptides.

**Methods:**

An ELISA controlling for these factors was used to measure the specific binding of serum IgG and IgM to phosphorylated (“pTau;” phosphorylated at Serine-199 and Serine-202) and non-phosphorylated (“non-pTau”) tau 196-207 in subjects with NCI, MCI, or AD (*n* = 10/group). Between-group differences in these antibody levels were evaluated for statistical significance, and correlations were examined in pooled data from all subjects between these antibody levels and subject age, global cognitive functioning, and NFT counts.

**Results:**

Specific IgG binding to pTau and non-pTau was detected in all subjects except for one NCI control. Specific IgM binding was detected to pTau in all subjects except for two AD patients, and to non-pTau in all subjects. Mean pTau IgG was increased in MCI subjects by 53% and 70% vs. AD and NCI subjects respectively (both *p* < 0.05), while no significant differences were found between groups for non-pTau IgG (*p* = 0.052), pTau IgM, or non-pTau IgM. Non-pTau IgG was negatively associated with global cognition (Spearman rho = −0.50).

**Conclusions:**

Specific binding of serum IgG and IgM to phosphorylated and non-phosphorylated tau may be present in older persons regardless of their cognitive status. Serum IgG to phosphorylated tau may be increased in individuals with MCI, but this unexpected finding requires confirmation. The approach used in this study to measure specific serum antibodies to phosphorylated tau should be useful for measuring antibodies to other post-translationally-modified proteins that are of relevance to neurodegenerative disorders.

## Background

Tau protein-containing neurofibrillary tangles (NFTs) and amyloid-β (Aβ)-containing plaques are the main pathological findings in the Alzheimer’s disease (AD) brain. Tau is found primarily in neurons where it binds to tubulin; this binding facilitates tubulin’s assembly into microtubules, permitting neurite extension and stabilization [1]. Although tau is minimally phosphorylated in adults without cognitive impairment, its phosphorylation increases (“hyperphosphorylation”) in AD [2]. Tau’s hyperphosphorylation reduces its binding to tubulin [3], resulting in decreased axonal transport of vesicles and organelles [4] which may cause neuronal damage. Hyperphosphorylated tau’s ability to sequester normal tau and other microtubule-associated proteins may also contribute to its neurotoxic effects [5, 6]. Tau’s hyperphosphorylation promotes its aggregation [7], which is clinically relevant because soluble oligomers may be tau’s most neurotoxic conformation [8, 9].

Since the amyloid hypothesis was proposed [10], lowering of brain Aβ has been the main focus of experimental AD therapies. But the failure of this approach to slow disease progression in large randomized clinical trials has increased interest in targeting of AD’s tau pathology. Tau aggregates are present in the AD brain as NFTs, neuropil threads, and dystrophic neurites [11]. The significance of NFT formation in AD is uncertain because NFT-containing neurons can survive for decades [12].

In transgenic mouse models of tauopathy, tau pathology and its cognitive deficits can be prevented by vaccination with tau [13–16] or by administration of anti-tau antibodies [17–21]. These findings provided the rationale for a phase 1 AD trial of a tau vaccine whose results suggested that the vaccine was safe and immunogenic [22]. A phase 2 tau vaccination trial began in 2016. Studies have found that anti-tau antibodies can impair tau’s aggregation [23], facilitate microglial uptake and degradation of pathological tau aggregates [24], and inhibit the neurotoxic effects and neuron-to-neuron spreading of paired helical filament (PHF)-tau [25, 26]. Some anti-tau antibodies may also inhibit tau phosphorylation [27, 28].

The extent to which serum anti-tau antibodies are produced in older individuals without apparent cognitive impairments, and whether their levels of these antibodies differ from those in individuals with MCI or AD, are unclear. A recent study [29] evaluated peripheral blood B lymphocytes from 120 healthy subjects for reactivity to a panel of phosphorylated tau peptides. Fifty-two anti-tau antibodies were identified, 26 of which appeared to be specific for phosphorylated tau. These results were suggested to represent an ongoing immune response against tau in these healthy individuals. Rosenmann et al. [30], measuring IgG and IgM to phosphorylated and non-phosphorylated tau, reported increased IgM levels to phosphorylated tau in AD patients compared to healthy subjects. Bartos et al. [31], measuring serum IgG antibodies to bovine tau protein (which would have contained phosphorylated amino acid residues, unlike recombinant human tau which is non-phosphorylated [32]), found similar levels between AD patients, individuals with other dementias, control subjects with neuroinflammatory conditions, and healthy subjects. Although both studies used enzyme-linked immunosorbent assays (ELISAs), neither study distinguished between antibody’s specific binding to tau and its polyvalent binding, which is also specific [33] but includes binding to multiple antigens such as surface epitopes on ELISA plate wells or wells coated with irrelevant proteins. We reported that polyvalent binding of human immunoglobulins (which we referred to as nonspecific binding) can result in overestimation of specific antibody levels measured by ELISA [34–36]. The study by Rosenmann et al. [30] also did not account for the possibility that the serum antibody binding which was detected to phosphorylated tau (tau peptide 195-213, phosphorylated at tau 202/205) could have been due, at least in part, to antibody binding to non-phosphorylated epitopes on the tau peptide. Using an ELISA which controlled for polyvalent binding, we detected specific antibodies (IgG) to non-phosphorylated tau (recombinant human tau-441, 2N4R) in three intravenous immunoglobulin (IVIG) products [35]. However, in a later study using an ELISA which also controlled for binding to non-phosphorylated epitopes on phosphorylated tau, we found specific antibodies to a phosphorylated tau peptide in only three of six IVIG preparations [37]. This result raised questions about the presence of specific anti-phospho-tau antibodies in healthy individuals, leading to the present investigation. This was an exploratory study whose primary goal was to determine the frequency of specific serum IgG and IgM binding to a phosphorylated and non-phosphorylated tau peptide (the same tau peptide that was used in our earlier IVIG study [37]) in individuals with NCI, MCI, or AD. The secondary goal in this study was to compare the specific anti-tau antibody levels between these diagnostic groups. We used an ELISA which controlled for polyvalent antibody binding and for antibody binding to non-phosphorylated epitopes on phosphorylated tau.

## Methods

### Study subjects

Serum samples were obtained from the Rush Alzheimer’s Disease Center (Chicago, IL, USA) from individuals participating in the Religious Orders Study [38] or the Rush Memory and Aging Project [39]. These are community-based studies of aging and AD which were approved by the Institutional Review Board of Rush University Medical Center. The clinical diagnoses proximate to death, based on review of clinical data blinded to neuropathologic findings, for the subjects in the present study were NCI, MCI, or AD (*n* = 10/group). AD patients had no other apparent cause of cognitive impairment. A global cognitive functioning score [40] was calculated for all subjects from 19 tests covering five domains: episodic memory, semantic memory, working memory, perceptual orientation, and perceptual speed. Raw scores for each test were converted to z-scores, then averaged. NFT counts were obtained during post-mortem neuropathological examination; these were mean values for NFT cortical density (per mm^2^) detected by immunocytochemical staining with monoclonal antibody AT8 [41] in the hippocampus and entorhinal, midfrontal, inferior temporal, angular, calcarine, anterior cingulate, and superior frontal cortices. Mean counts were transformed by square root to produce more normally distributed data. The study enrolled only persons without known dementia, therefore all subjects signed an informed consent and an Anatomical Gift Act for organ donation. De-identified serum samples were sent from the Rush Alzheimer’s Disease Center to the Neurology Research Laboratory at Beaumont Hospital-Royal Oak (parent organization: Beaumont Health, Royal Oak, MI, USA) where anti-tau antibodies were measured. The present research study was given exempt status by the Institutional Review Board of Beaumont Health.

### Measurement of specific anti-tau antibodies

Anti-tau antibodies in NCI, MCI, and AD sera were measured by ELISA using methods similar to those that we reported previously for measuring antibodies to phosphorylated tau in IVIG [37]. Tau peptide 196-207, phosphorylated (“pTau”) or non-phosphorylated (“non-pTau”) at Serine-199 and Serine-202, was used as the antigen for antibody detection. These peptides were chemically synthesized by the Tufts University Core Facility (Tufts Medical School, Boston, MA, USA), using Fmoc (Fluorenylmethyloxycarbonyl chloride) - protected amino acids and Fastmoc chemistry on an ABI 431 Peptide Synthesizer (Applied Biosystems, Foster City, CA). Serum samples were diluted 1:100 in phosphate-buffered saline (0.01 M, pH 7.2) with 1% BSA and 0.5% Tween-20 (hereafter, PBS-T-BSA) and evaluated in quadruplicate for anti-tau IgG and in triplicate for anti-tau IgM in wells coated with pTau, non-pTau, or BSA. To account for polyvalent antibody binding [33], the optical density [OD] values that developed in wells in which serum samples were incubated on BSA-coated wells were subtracted from the OD values of the wells in which the samples were incubated on pTau- and non-pTau-coated wells. ELISAs measuring IgG binding to pTau or non-pTau included a standard curve with rabbit anti-pS199 polyclonal antibody (GenScript, Piscataway, NJ, USA) diluted four-fold in PBS-T-BSA from 1:62.5 (8000 ng/mL) to 1:16,000 (31.25 ng/mL). No standard curve was included in ELISAs measuring anti-tau IgM because commercial anti-tau IgM was not available; as positive controls, GenScript’s rabbit anti-pS199 antibody (phospho-tau specific; 1:160 in PBS-T-BSA) and AnaSpec’s rabbit anti-tau 194-214 IgG (AnaSpec Inc., Fremont, CA, USA; non-phospho-tau specific; 1:160 in PBS-T-BSA) were incubated on wells coated with 50 μg/mL of pTau, non-pTau, or BSA (three wells per condition). As a negative control, two wells per condition in IgG ELISAs and three wells per condition in IgM ELISAs were incubated with PBS-T-BSA instead of NCI, MCI, or AD sera. Plates were developed by incubation with biotinylated goat anti-human IgG (Jackson ImmunoResearch Laboratories Inc., West Grove, PA, USA) or biotinylated goat anti-human IgM (Sigma-Aldrich Co., St. Louis, MO, USA) followed by streptavidin-alkaline phosphatase (Life Technologies, Rockford, IL, USA) and para-nitrophenol phosphate (Sigma-Aldrich) as reported previously [35, 37]. IgG plates were incubated at room temperature for 2 hr., then read at 405 nm. The standard curve typically reached an OD value between 0.5 - 0.7. IgM plates were incubated at room temperature and read until OD values for the non-pTau-coated wells which had been incubated with rabbit anti-tau 194-214 antibody reached 1.0; this occurred after about 15 min. Mean OD values of wells in which PBS-T-BSA was incubated on pTau, non-pTau, or BSA were subtracted from mean OD values of wells in which serum samples were incubated on tau peptides or BSA. Ratios for IgG and IgM binding of each serum sample to pTau and non-pTau (hereafter, “pTau antibody ratios” and “non-pTau antibody ratios”) were calculated as follows:

antibody binding to pTau peptide:

$$ {\displaystyle \begin{array}{l}\frac{\mathrm{OD}\ \mathrm{for}\  \mathrm{binding}\  \mathrm{to}\  \mathrm{pTau}\  \mathrm{peptide}\hbox{--} \mathrm{OD}\ \mathrm{for}\  \mathrm{binding}\  \mathrm{to}\ \mathrm{BSA}}{\mathrm{OD}\ \mathrm{for}\  \mathrm{binding}\  \mathrm{to}\  \mathrm{non}\hbox{-} \mathrm{pTau}\  \mathrm{peptide}\hbox{--} \mathrm{OD}\ \mathrm{for}\  \mathrm{binding}\  \mathrm{to}\ \mathrm{BSA}}\\ {}\mathrm{antibody}\  \mathrm{binding}\  \mathrm{to}\  \mathrm{non}\hbox{-} \mathrm{pTau}\  \mathrm{peptide}:\frac{\mathrm{OD}\ \mathrm{for}\  \mathrm{binding}\  \mathrm{to}\  \mathrm{non}\hbox{-} \mathrm{pTau}\  \mathrm{peptide}}{\mathrm{OD}\ \mathrm{for}\  \mathrm{binding}\  \mathrm{to}\ \mathrm{BSA}}\end{array}} $$

Antibody binding ratios greater than 1.0 were accepted as evidence for the presence of specific anti-tau antibodies, while ratios less than 1.0 were not considered to indicate specific anti-tau antibodies and were recorded as 1.0 for statistical purposes.

### Statistics

Continuous variables whose distribution was approximately normal were summarized with mean and standard deviation while other continuous variables were summarized with median and range. Model diagnostics were examined to assess the reasonableness of the assumptions of normality and equal variances which needed to be satisfied in order to perform analysis of variance (ANOVA). Overall tests of group differences used ANOVA or Kruskal-Wallis tests depending on the reasonableness of the procedure assumptions; either Tukey or Dwass, Steel and Critchlow-Fligner multiple comparison procedures respectively were used if needed to determine the location of pairwise differences. Spearman’s rank-order correlation coefficient (rho) was used to examine associations between variables. *P*-values < 0.05 were considered to be statistically significant. Analyses used The SAS System for Windows version 9.3 (Cary, NC). Graphs were generated by Minitab release 14 (State College, PA).

## Results

### Study subjects

Summary statistics for the study subjects are shown in Table 1. The groups were well balanced for age and gender but differed in global cognitive functioning scores. The AD group had lower scores than the other two groups for all cognitive domains; although median cognitive scores for all domains were lower for MCI than for NCI subjects, the differences were statistically significant only for episodic memory (data not shown). AD patients had MMSE scores ranging from 13 to 20 and clinical diagnoses of mild to moderate AD, while MMSE scores in MCI and NCI subjects ranged from 23 to 29 and from 27 to 30 respectively. Mean NFT counts were highest in AD subjects.

**Table 1:** Demographic, clinical, and neuropathologic characteristics for NCI, MCI, and AD subjects

Variables	NCI (*n* = 10)	MCI (*n* = 10)	AD (*n* = 10)
Age, yrs	88.8 ± 4.8 (81.9 – 96.6)	89.8 ± 3.7 (84.3 – 96.4)	88.5 ± 3.0 (83.6 – 92.0)
% Males	40	40	40
Global cognitive functioning score	0.26 ([−0.15] – [1.12])	−0.21 ([−0.54] – [0.26])	−1.31 ([−3.03] – [−1.08])
MMSE score	28.8 ± 1.1 (27 – 30)	26.1 ± 2.2 (23 – 29)	16.9 ± 2.3 (13 – 20)
NFT counts	3.50 ± 2.68 (0.57 – 8.03)	3.81 ± 2.53 (0.80 – 7.18)	8.14 ± 5.87 (2.13 – 17.55)

Summary statistics for age, MMSE scores, and NFT counts are shown as mean ± SD (top line) and range (bottom line), while global cognitive functioning scores are shown as median (top line) and range (bottom line). (*NCI* No cognitive impairment, *MCI* Mild cognitive impairment, *AD* Alzheimer’s disease, *MMSE* Mini Mental State Examination, *NFT* Neurofibrillary tangles)

### Binding of serum IgG and IgM to pTau peptide in NCI, MCI, and AD subjects

**Fig. 1:**
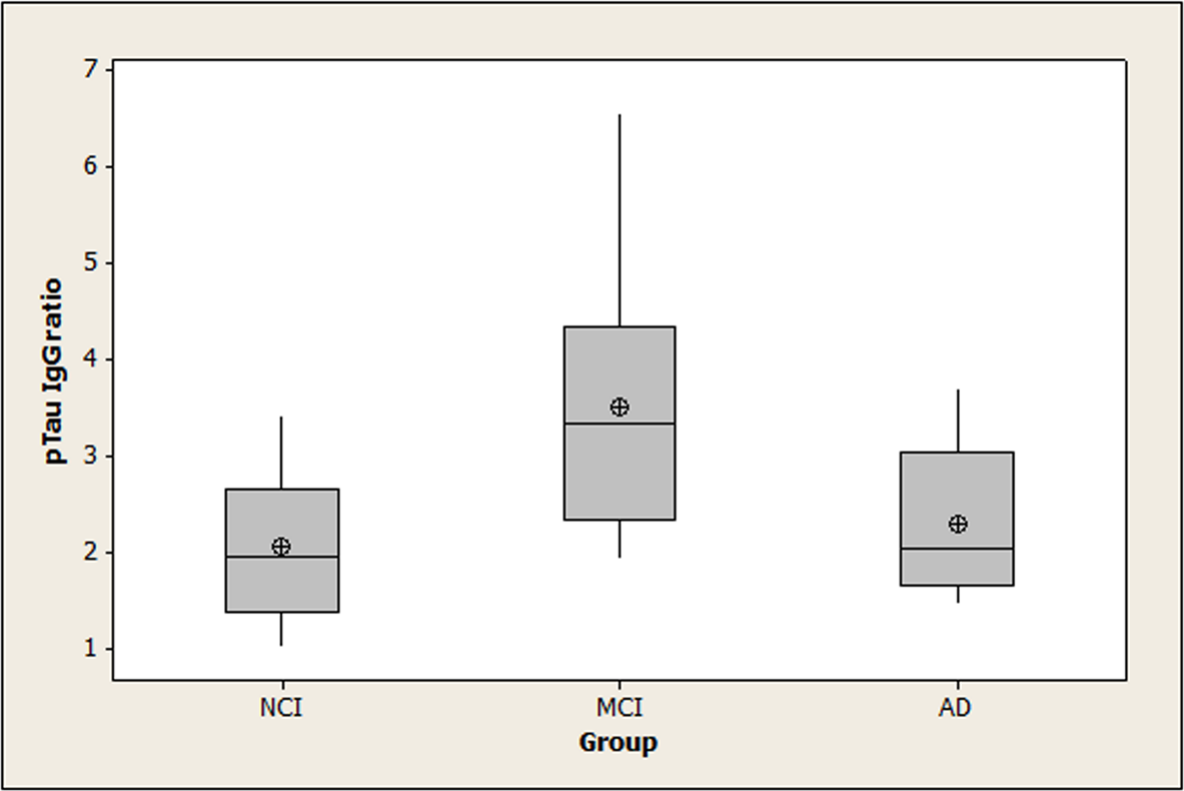
Binding of serum IgG to pTau peptide in NCI, MCI, and AD subjects. Means (circle), medians (line through center of box), upper and lower quartiles (upper and lower borders of box, respectively), most extreme non-outlier values (lines extending from box), and outliers (asterisks) are shown for pTau IgG ratios in serum samples from NCI, MCI, and AD subjects. “pTau IgG ratio” is the ratio of a serum sample’s specific IgG binding to phosphorylated tau peptide-coated wells divided by its specific IgG binding to non-phosphorylated tau peptide-coated wells: $\frac{\mathrm{OD}\ \mathrm{for}\ \mathrm{IgG}\ \mathrm{binding}\  \mathrm{to}\  \mathrm{pTau}\  \mathrm{peptide}\hbox{--} \mathrm{OD}\ \mathrm{for}\ \mathrm{IgG}\ \mathrm{binding}\  \mathrm{to}\ \mathrm{BSA}}{\mathrm{OD}\ \mathrm{for}\ \mathrm{IgG}\ \mathrm{binding}\  \mathrm{to}\  \mathrm{non}\hbox{-} \mathrm{pTau}\  \mathrm{peptide}\hbox{--} \mathrm{OD}\ \mathrm{for}\ \mathrm{IgG}\ \mathrm{binding}\  \mathrm{to}\ \mathrm{BSA}}$ ANOVA found evidence of group differences (*p* = 0.008), with significance at the 0.05 level for the pairwise comparisons between the MCI group and each of the other groups. (NCI = no cognitive impairment; MCI = mild cognitive impairment; AD = Alzheimer’s disease; pTau IgG = specific IgG binding to tau 196-207 phosphorylated at Serine-199 and Serine-202)

**Fig. 2:**
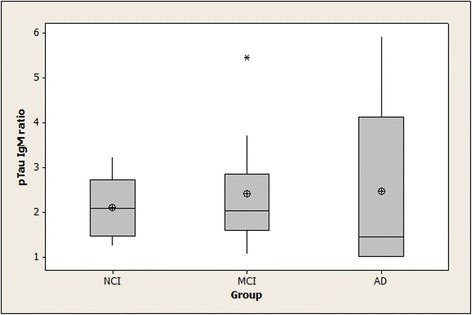
Binding of serum IgM to pTau peptide in NCI, MCI, and AD subjects. Means (circle), medians (line through center of box), upper and lower quartiles (upper and lower borders of box, respectively), most extreme non-outlier values (lines extending from box), and outliers (asterisks) are shown for pTau IgM ratios in serum samples from NCI, MCI, and AD CTL subjects. “pTau IgM ratio” is the ratio of a serum sample’s specific IgM binding to phosphorylated tau peptide-coated wells divided by its specific IgM binding to non-phosphorylated tau peptide-coated wells: $\frac{\mathrm{OD}\ \mathrm{for}\ \mathrm{IgM}\ \mathrm{binding}\  \mathrm{to}\  \mathrm{pTau}\  \mathrm{peptide}\hbox{--} \mathrm{OD}\ \mathrm{for}\ \mathrm{IgM}\ \mathrm{binding}\  \mathrm{to}\ \mathrm{BSA}}{\mathrm{OD}\ \mathrm{for}\ \mathrm{IgM}\ \mathrm{binding}\  \mathrm{to}\  \mathrm{non}\hbox{-} \mathrm{pTau}\  \mathrm{peptide}\hbox{--} \mathrm{OD}\ \mathrm{for}\ \mathrm{IgM}\ \mathrm{binding}\  \mathrm{to}\ \mathrm{BSA}}$ The overall test of between-group differences was not statistically significant (*p* = 0.79). (NCI = no cognitive impairment; MCI = mild cognitive impairment; AD = Alzheimer’s disease; pTau IgM = specific IgM binding to tau 196-207 phosphorylated at Serine-199 and Serine-202)

pTau IgG antibody ratios > 1.0 were found for all study subjects except for one member of the NCI group. The data were distributed reasonably normally for pTau IgG ratios but not for pTau IgM ratios. The mean pTau IgG ratio was increased in MCI subjects by 53% and 70% vs. AD and NCI subjects respectively; there was evidence of between-group differences (*p* = 0.008), with statistical significance for the pairwise comparisons between the MCI group and the other groups (MCI vs. AD: *p* = 0.033; MCI vs. NCI: *p* = 0.01). pTau IgM ratios >1.0 were detected for all NCI and MCI subjects and for eight of the 10 AD patients, with no statistically significant differences in the ratios between the groups (*p* = 0.79). Summary statistics for the pTau IgG and pTau IgM ratios are shown in Figs. 1 and 2.

### Binding of serum IgG and IgM to non-pTau peptide in NCI, MCI, and AD subjects

**Fig. 3:**
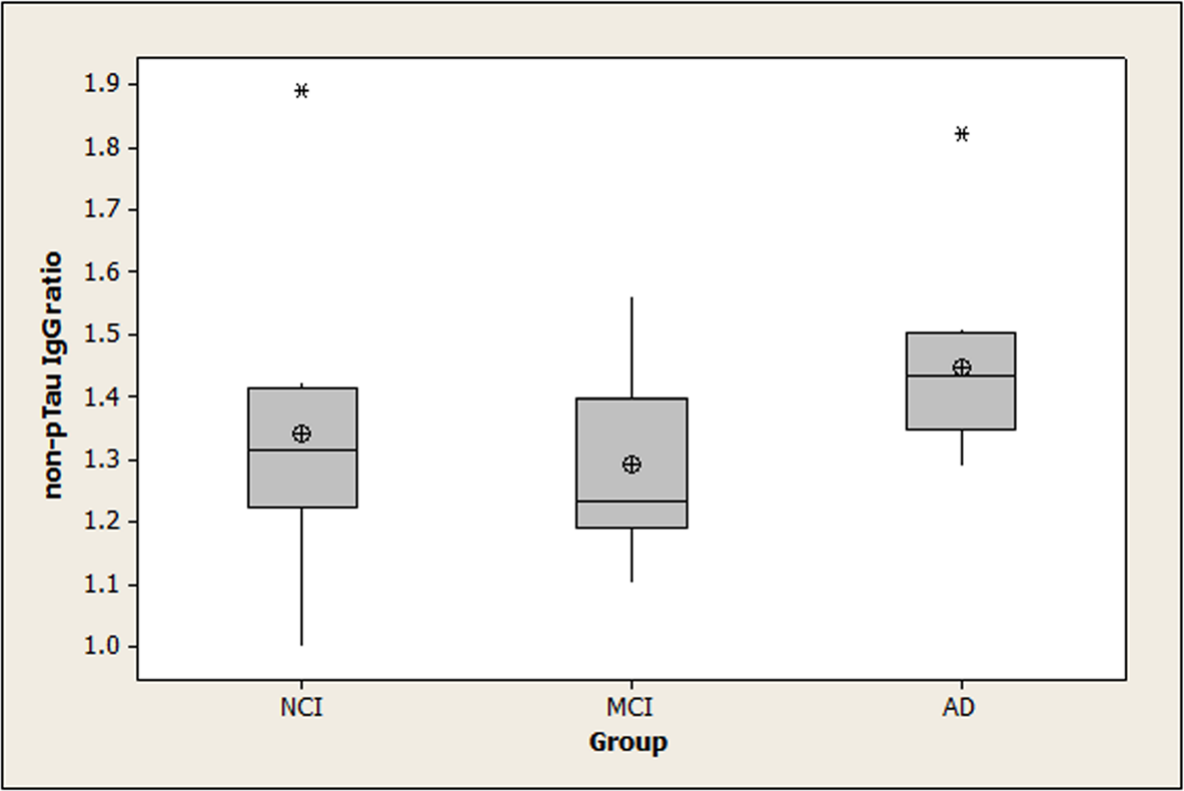
Binding of serum IgG to non-pTau peptide in NCI, MCI, and AD subjects. Means (circle), medians (line through center of box), upper and lower quartiles (upper and lower borders of box, respectively), most extreme non-outlier values (lines extending from box), and outliers (asterisks) are shown for non-pTau IgG ratios in serum samples from NCI, MCI, and AD subjects. “non-pTau IgG ratio” is the ratio of a serum sample’s IgG binding to non-phosphorylated tau peptide-coated wells divided by its IgG binding to $\mathrm{BSA}\hbox{-} \mathrm{coated}\  \mathrm{wells}:\frac{\mathrm{OD}\ \mathrm{for}\ \mathrm{IgG}\ \mathrm{binding}\  \mathrm{to}\  \mathrm{non}\hbox{-} \mathrm{pTau}\  \mathrm{peptide}}{\mathrm{OD}\ \mathrm{for}\ \mathrm{IgG}\ \mathrm{binding}\  \mathrm{to}\ \mathrm{BSA}}$ The *p*-value for the between-group differences for the antibody ratios was 0.052. (NCI = no cognitive impairment; MCI = mild cognitive impairment; AD = Alzheimer’s disease; non-pTau IgG = specific IgM binding to non-phosphorylated tau 196-207)

**Fig. 4:**
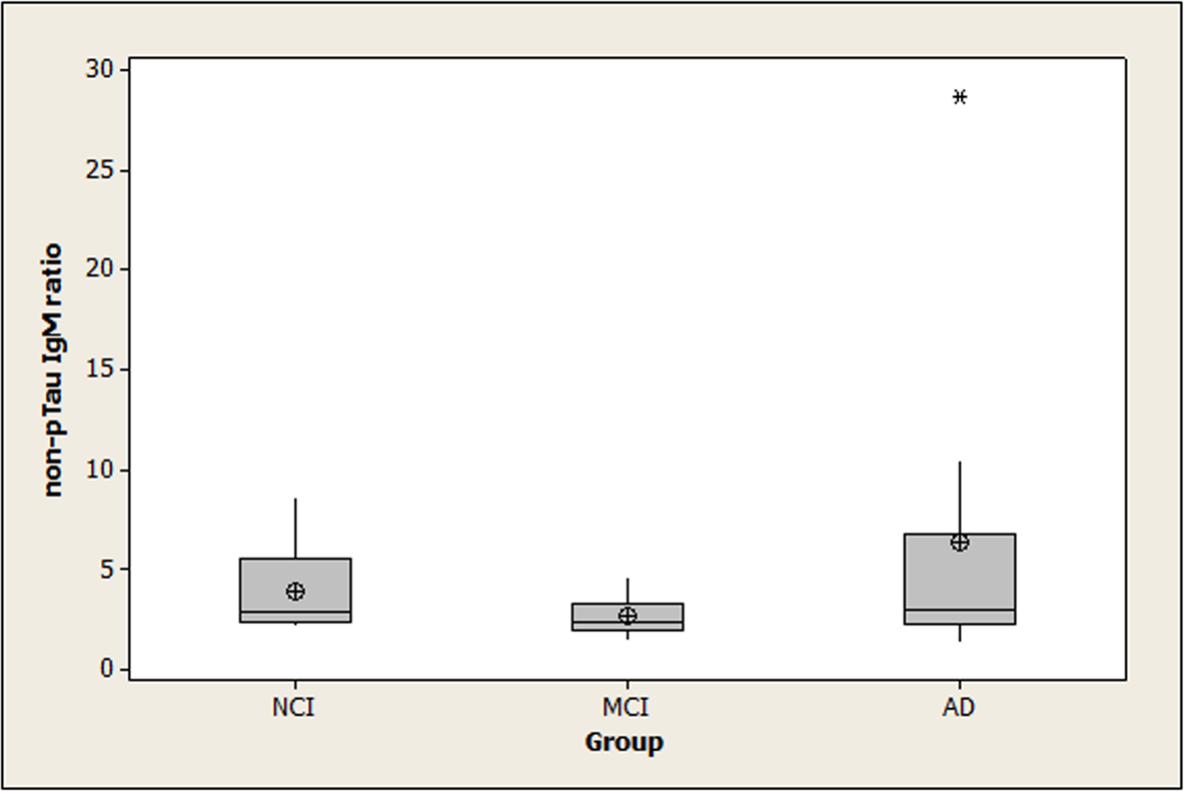
Binding of serum IgM to non-pTau peptide in NCI, MCI, and AD subjects. Means (circle), medians (line through center of box), upper and lower quartiles (upper and lower borders of box, respectively), most extreme non-outlier values (lines extending from box), and outliers (asterisks) are shown for non-pTau IgM ratios in serum samples from NCI, MCI, and AD subjects. “non-pTau IgM ratio” is the ratio of a serum sample’s IgM binding to non-phosphorylated tau peptide-coated wells divided by its IgM binding $\mathrm{to}\ \mathrm{BSA}\hbox{-} \mathrm{coated}\  \mathrm{wells}:\frac{\mathrm{OD}\ \mathrm{for}\ \mathrm{IgM}\ \mathrm{binding}\  \mathrm{to}\  \mathrm{non}\hbox{-} \mathrm{pTau}\  \mathrm{peptide}}{\mathrm{OD}\ \mathrm{for}\ \mathrm{IgM}\ \mathrm{binding}\  \mathrm{to}\ \mathrm{BSA}}$ The between-group differences did not achieve statistical significance (*p* = 0.26). (NCI = no cognitive impairment; MCI = mild cognitive impairment; AD = Alzheimer’s disease; non-pTau IgM = specific IgM binding to non-phosphorylated tau 196-207)

Non-pTau IgG ratios > 1.0 were found for all subjects except for one member of the NCI group (the same subject who lacked detectable specific anti-pTau IgG). The data for the non-pTau IgG ratios were abnormally distributed, necessitating the use of a non-parametric procedure (the Kruskal-Wallis test) for examining statistical significance of the overall differences between groups. The *p*-value for overall differences between groups for the non-pTau IgG ratios was 0.052, with the highest median value in the AD group. Non-pTau IgM ratios > 1.0 were found for all subjects. These ratios were also abnormally distributed. Kruskal-Wallis testing found no differences between the groups (*p* = 0.26). Summary statistics for the non-pTau IgG and IgM ratios are shown in Figs. 3 and 4.

### Correlations between tau antibody binding ratios, subject age, cognitive scores, and NFT counts

Correlations were examined, in pooled data from all subjects, between the four tau antibody ratios, and between each ratio and subject age, global cognitive scores, and NFT counts. Spearman’s rho values and their associated *p*-values are shown in Table 2. A negative correlation was found between the pTau and non-pTau IgM ratios (rho = −0.62; *p* = 0.0002). The associations between the other antibody ratios were weak (rho absolute values < 0.20). Age was positively associated with the non-pTau IgG ratio (rho = 0.55; *p* = 0.002). Global cognitive functioning scores and the non-pTau IgG ratio were negatively correlated (rho = −0.50; *p* = 0.005). Correlations between tau antibody ratios and NFT counts were weak (Spearman rho absolute values < 0.25).

**Table 2:** Correlations between tau antibody binding ratios, subject age, cognitive scores, and NFT counts using all study subjects

	pTau IgG	non-pTau IgM	pTau IgM	Age	Global Cognition	NFT counts
Non-pTau IgG	−0.14	−0.01	0.12	0.55	−0.50	0.14
	0.47	0.95	0.51	0.002	0.005	0.46
pTau IgG		−0.11	0.08	0.09	0.13	0.20
		0.57	0.69	0.62	0.49	0.30
non-pTau IgM			−0.62	−0.08	0.05	0.24
			0.0002	0.66	0.81	0.20
pTau IgM				0.23	−0.05	−0.13
				0.22	0.78	0.50

Correlations in pooled data from all subjects are shown as Spearman’s rho values (top line) and their associated p-values (bottom line). The pTau IgM and non-pTau IgM ratios were negatively correlated, as were global cognitive functioning scores and the non-pTau IgG ratio. Subject age was positively associated with the non-pTau IgG ratio. (NFT = neurofibrillary tangles; pTau IgG = ratio of IgG binding to tau 196-207 phosphorylated at Serine-199 and Serine-202; non-pTau IgG = ratio of IgG binding to non-phosphorylated tau 196-207; pTau IgM = ratio of IgM binding to tau 196-207 phosphorylated at Serine-199 and Serine-202; non-pTau IgM = ratio of IgM binding to non-phosphorylated tau 196-207)

## Discussion

This was an exploratory study to examine binding of serum IgG and IgM from NCI, MCI, and AD subjects to a phosphorylated and non-phosphorylated tau peptide. Antibody binding ratios > 1.0 were accepted as evidence for the presence of specific anti-tau antibodies. The main goal of the study was to determine the frequency of these antibodies in the subjects in each of the diagnostic groups. Specific serum antibodies to both phosphorylated and non-phosphorylated tau were detected in most subjects regardless of cognitive status. The secondary goal of the study was to compare specific anti-tau antibody levels between the three diagnostic groups. Increased specific IgG binding to the phosphorylated tau peptide (an increase in the pTau IgG ratio) was detected in MCI subjects compared to NCI and AD subjects. This finding was unexpected, and requires confirmation. Although NFT pathology can be present in individuals with MCI [42–44], the mean NFT count in our MCI group was only slightly increased compared to that in our NCI group, and NFT counts in the brain regions represented by the mean NFT counts appeared similar between MCI and NCI subjects (data not shown). We did not find statistically significant differences between the three diagnostic groups for the other anti-tau antibody ratios, although we found a *p*-value of 0.052 for the overall differences between groups for the non-pTau IgG ratio. Given our relatively small group sizes, we cannot rule out the possibility of group differences for the non-pTau IgG, pTau IgM, and non-pTau IgM ratios.

Our correlational analyses produced some interesting findings. We found a negative correlation (rho = −0.62) between the pTau IgM and non-pTau IgM ratios. IgM is produced during the initial humoral (antibody) immune response to an antigen; this response peaks, then decreases more rapidly than the anamnestic response which occurs upon repeated exposure to an antigen [45]. Antibodies produced during the anamnestic response are mainly IgG, but some IgM is also produced [46]. Perhaps, as brain tau pathology increases (and tau that is increasingly phosphorylated leaks across the blood brain barrier [BBB]), the immune system may come into contact to a greater extent with phosphorylated rather than non-phosphorylated tau; this could result in increased production of anti-pTau antibodies as levels of antibodies to non-pTau decrease. However, this would not explain why a negative correlation was not also found between the pTau IgG and non-pTau IgG ratios.

We found a positive association between subject age and the non-pTau IgG ratio (rho = 0.55). This was unlikely to have contributed to the negative correlation between non-pTau IgG levels and global cognitive functioning (rho = −0.50) because age distribution was similar in our groups, while global cognition differed between groups. The negative correlation between non-pTau IgG and global cognitive functioning suggests that decreased cognition in some subjects may have been associated with an increase in their non-pTau IgG. However, the median non-pTau IgG ratio was only 16% higher for AD than for NCI subjects, and it was slightly lower for MCI than for NCI subjects (Fig. 3), so the relevance of this correlation is unclear.

The effects of serum anti-tau antibodies on the development and neuropathological progression of AD are unknown. Immunoglobulins are able to enter neurons [13, 47, 48], so if serum anti-tau antibodies gain limited access to the brain, they might reduce intraneuronal levels of aggregated and/or phosphorylated tau [48–50]. Tau is also present extracellularly in “tombstone tangles” and during spreading of aggregated or misfolded tau between neurons [51–53], so these antibodies might slow the spreading of tau pathology and/or promote microglial clearance of extracellular tau aggregates [25]. Although serum antibodies should not cross an intact BBB, an age-dependent progressive loss of BBB integrity in the hippocampus has been reported in NCI subjects [54], and BBB permeability increases in some MCI and AD subjects [55–57]. Therefore serum anti-tau antibodies could reduce the neurotoxicity of pathological tau conformations in these individuals.

This study has some limitations. It was an exploratory study, because we did not know if specific anti-tau antibodies would be detectable in our subjects; we therefore limited our group sizes to 10 subjects each. The lack of statistical significance for the between-group differences for non-pTau IgG (*p* = 0.052), whose largest median value was in the AD group, could have been due to insufficient statistical power. We could not perform a post-hoc power analysis to estimate appropriate group sizes needed for reasonable statistical power for future comparisons of non-pTau IgG between NCI, MCI, and AD subjects, because the assumptions for ANOVA did not appear to be satisfied for our non-pTau IgG data. Finally, we do not know if results similar to those we found in this study would have been obtained with different non-phosphorylated or phosphorylated tau peptides.

## Conclusions

Specific antibodies to phosphorylated and non-phosphorylated tau may be present in sera from older individuals regardless of their cognitive status. Specific serum IgG binding to phosphorylated tau may be increased, for unknown reasons, in individuals with MCI, but this finding requires confirmation. The approach used in this study to measure specific serum antibodies to phosphorylated tau should be of value for measuring antibodies to other neurodegeneration-related proteins that are post-translationally modified, such as α-synuclein in Parkinson’s disease [58] and huntingtin in Huntington’s disease [59].

## Abbreviations

Aβ: Amyloid-β
AD: Alzheimer’s disease
ANOVA: Analysis of variance
BBB: Blood-brain barrier
BSA: Bovine serum albumin
ELISA: Enzyme-linked immunosorbent assay
IVIG: Intravenous immunoglobulin
MCI: mild cognitive impairment
NCI: No cognitive impairment
NFTs: Neurofibrillary tangles
non-pTau: Non-phosphorylated tau
OD: Optical density
PBS: Phosphate-buffered saline
pTau: Phosphorylated tau
